# Book Notices

**Published:** 1867-06

**Authors:** 


***Book Notices.***

Appreciative notices of the books acknowledged as received, in the last JOURNAL, are crowded out of the present issue by the unexpected space occupied by communicated articles. They are each of them worth adding to the library, and, more than this, of being thoroughly read, marked, and inwardly digested. Next month, we shall speak of them individually and in detail.

